# 

**Published:** 1872-01

**Authors:** 


### Contents of January Number.

### ORIGINAL DEPARTMENT.

ART. I.—Medical Society of the County of Albany, Semi-monthly Meeting, Dec. 13th, 1871. Reported by James S. Bailey, M. D. .... 201
ART. II.—Fractures treated at the Buffalo General Hospital. By C. C. F. Gay, M. D. .... 207
ART. III.—On the recent advances in the Theory of Vision. By H. Helmboltz. Translated by F. W. Abbott, M. D. .... 209
ART. IV.—Periodical Headache. A new method of Curative Treatment. By F. Bradnack, M. D. .... 220
ART. V.—A Case of Ovariotomy. By J. S. Dolson, M. D. .... 227
ART. VI.—Abscess of Temporal Region from Gunshot injury. Extraction of ball after four years lodgment behind Zygomatic Arch. By J. W. Southworth, M. D. .... 229

### CORRESPONDENCE.

Primary Board appointed for the purpose of examining young men before entering the Study of Medicine .... 230
Genesee County Medical Society .... 231

### MISCELLANEOUS.

On Arterial Transfusion of Blood .... 2[illegible]2

### EDITORIAL DEPARTMENT.

Annual Meeting of the Erie County Medical Society. Reception of a Portrait of Dr. Levi J. Ham .... 233
Vaccination and Small Pox .... 234
BOOKS REVIEWED:
Practical Treatise on Fractures and Dislocations. By Frank Hastings Hamilton, A.M., M.D., L.L.D. .... 235
A Text Book of Pathological Histology; an introduction to the study of Pathological Anatomy. By Dr. Edward Rindfleisch. Translated from the German by William C. Kloman, M. D., and E. T. Miles, M. D .... 236
Emergencies and how to treat them. By Jos. N. Howe, M.D. .... 236
The Druggists General Receipt Book, with Veterinary Formulary. By Henry Beasley .... 237
Disorders of the Nervous System in Childhood. By Chas. West, M. D. .... 237
Neumann's Hand Book of Skin Diseases. Translated from the German by Lucius D Buckley, M. D. .... 237
Functions and Disorders of the Reproductive Organs in Childhood, Youth, Adult Age and Advanced Life. By William Acton, M. R. C. S. .... 237
Restorative Medicine. By Thomas King Chambers, M. D. .... 238
American Medical Association, .... 238
Meeting of the New York State Medical Society, .... 239
Medical Department Harvard University, .... 239
Deaths from Chloroform, .... 239
The St. Louis Medical and Surgical Journal, .... 239
Prof. Crocq, of Brussels, on Cholera Contagion, .... 239
Books and Pamphlets Received, .... 240

THE BUFFALO

Medical and Surgical Journal

